# Corticosteroid-Induced Mania After Previous Tolerance of Higher Doses

**DOI:** 10.7759/cureus.17719

**Published:** 2021-09-04

**Authors:** Reena Jasani, Jennifer W Deacon, Anthony Sertich

**Affiliations:** 1 Internal Medicine, University of Texas Southwestern Medical Center, Dallas, USA

**Keywords:** steroid adverse effects, systemic lupus erythromatosus, corticosteroid-induced mania, exogenous steroids, corticosteroid-induced psychosis

## Abstract

Corticosteroids have several widely documented adverse effects. However, there is no systematic study evaluating the frequency and associations of corticosteroid-induced mania. We report a case of corticosteroid-induced mania in a patient that previously tolerated higher doses of steroid therapy without neuropsychiatric symptoms. Although there is evidence suggesting a dose-dependent relationship, previous tolerance has not been proven to correlate with reduced frequency of developing mania or psychosis on the reintroduction of the medication. Therefore, patients restarting steroids should proceed carefully, even when receiving low doses.

## Introduction

For decades corticosteroids have been used to treat a number of medical diseases, ranging from local and acute to systemic and chronic conditions. By interfering with the migration of leukocytes through the endothelium of blood vessels, corticosteroids prevent leukocytes from reaching and responding to sites of tissue damage and infection, effectively suppressing inflammation [1]. Despite their efficacy for a variety of inflammatory conditions, corticosteroids are widely known to cause a range of adverse effects. Many of these effects have been extensively studied and reported, including hirsutism, obesity, neutrophilia, osteoporosis, and oral candidiasis [2]. However, the psychiatric side effects of steroids have not been evaluated as methodically and widely.

Of the various symptoms of corticosteroid-induced neuropsychiatric disorders, the most common are manic features including irritability, euphoria, pressured speech, hyperactivity, and distractibility [3,4]. Patients can also present with depression, hypomania, psychosis, panic attacks, agoraphobia, insomnia, catatonia, impaired memory, and obsessive-compulsive disorder [3]. While studies have not revealed accurate predictors of corticosteroid-induced neuropsychiatric symptoms, these symptoms have been found to be more common in females [3,5]. Further, patients with systemic lupus erythematosus (SLE) have been found to have a greater risk of developing corticosteroid-induced neuropsychiatric symptoms [3,5].

Existing research also suggests that there is a dose-dependent relationship between steroids and neuropsychiatric symptoms, with increased cases reported at higher doses [3,6]. We present a case of corticosteroid-induced mania in a patient that previously tolerated higher doses of steroid therapy without neuropsychiatric symptoms, highlighting the possibility for such side effects to develop on resumption rather than the introduction of steroid use.

## Case presentation

A 36-year-old African American woman with a past medical history of systemic lupus erythematosus (SLE), a recent novel coronavirus infection severe acute respiratory syndrome coronavirus 2 (SARS-CoV-2, COVID-19) not requiring supplemental oxygen and treated with intravenous steroids, and hypertension presented to the hospital with acute nausea, vomiting, and fever. The patient had recently been admitted for fatigue from SLE and was discharged on hydroxychloroquine, mycophenolate, and prednisone taper to a rehabilitation facility to continue physical therapy one month prior. On discharge from the rehabilitation facility, the patient reported, she was not prescribed prednisone. She reported she had been tapering down every two weeks from a starting dose of 80 mg and had reached 30 mg daily. After a few days on 30 mg, she ran out of pills and was unable to get a new prescription. After she was without prednisone for three days, she started having nausea and vomiting and was unable to tolerate any oral intake. She endorsed diarrhea and fatigue but denied subjective fever, chills, weight loss, abdominal pain, melena, hematochezia, hematemesis, joint pain, joint swelling, oral sores, hair loss, and facial rash.

The patient had no past psychiatric history. The patient’s medications were amlodipine, calcium carbonate-vitamin D3, hydroxychloroquine, mycophenolate, nystatin powder, pantoprazole, and prednisone (the last dose was three days prior to admission). The patient had no known drug allergies. Her only past surgery was a cesarean section. The patient reported working virtually as a court clerk and performing all her activities of daily living independently. Due to generalized weakness after her hospital admission for a lupus flare and COVID-19 infection, she reported using a walker when walking long distances. She denied using tobacco, alcohol, and any illicit drugs. She reported a family history of hypertension in multiple family members.

On physical examination, her temperature was 38.9 °C (102.0 °F), blood pressure was 146/97 mm Hg, heart rate was 111 beats per minute, respiratory rate was 18 breaths per minute, and oxygen saturation was 94%. The patient appeared tired but was alert, oriented, and well-developed. The rest of the exam was insignificant.

Pertinent laboratory data on admission are included in Table 1. Transaminases, bilirubin, and lipase were all within normal limits. Due to concerns for sepsis, the patient was admitted to the inpatient hospital medicine service, where she was restarted on her home prednisone at 30 mg daily and empirically started on ceftriaxone. Workup revealed a morning cortisol level of 3.0 mcg/dL and negative blood and urine cultures. The patient’s symptoms improved on steroids, and her symptoms were attributed to adrenal insufficiency.

**Table 1:** Laboratory data on admission. Abnormal lab values are indicated with (H) for higher or (L) for lower than the normal range for the lab test. dsDNA: double-stranded deoxyribonucleic acid.

Lab test	Result
White blood cell count	3.88 × 10^9^ cells/L (L)
Hemoglobin	6.6 g/dL (L)
Hematocrit	21.0% (L)
Reticulocyte count	1.6%
Haptoglobin	<5 mg/dL (L)
C-reactive protein	6.0 mg/dL (H)
Erythrocyte sedimentation rate	28 mm/hr (H)
C3 complement	73 mg/dL (L)
C4 complement	18 mg/dL
dsDNA antibody	Negative (previously positive)
Creatinine	1.08 mg/dL (H)
Urinalysis	3+ Protein, large blood

On her third day of admission, the patient became tachycardic and altered with elevated mood, decreased need for sleep, distractibility, hyper-religiosity, and inappropriate smiling and laughing. Due to concern for corticosteroid-induced mania, she was started on valproic acid 500 mg twice a day and clonazepam 1 mg at bedtime as needed for insomnia. Her prednisone was not discontinued due to her initial presentation of adrenal insufficiency but was decreased to 20 mg daily. Physical exam revealed no focal neurologic deficits. CT and MRI of the brain showed no acute abnormalities and no evidence of lupus cerebritis. Lumbar puncture showed low protein, elevated lymphocytes, and immunoglobulin G within normal limits. While cerebrospinal fluid analysis showed a lymphocytic pleocytosis, the low protein, lack of focal neurologic deficits on an exam, unremarkable MRI, and acute onset of symptoms following steroid resumption were more consistent with a diagnosis of substance-induced mood disorder rather than aseptic meningitis or other neurologic manifestations of lupus.

The patient returned to her baseline mental status with valproic acid within 24 hours. As she had previously been on 80 mg of prednisone, the dose was increased from 30 mg to 40 mg daily to allow for a more gradual steroid taper and prevent adrenal insufficiency. She was discharged with the plan of decreasing her valproic acid dose in parallel with her steroid taper. Although the goal was to discontinue valproic acid after she was off steroids, the patient ran out of valproic acid three months after discharge while still on prednisone (15 mg daily). She had been referred to psychiatry upon discharge to manage her valproic acid taper and was seeing a psychiatrist. However, she was not given refills at her previous psychiatry appointment for unknown reasons. Although she had a psychiatry appointment scheduled during the same month that she ran out of valproic acid, in a visit to the emergency department two months later, she reported she had not resumed her valproic acid. During that encounter, she reported she had not experienced further psychiatric symptoms while off valproic acid and had continued her steroid taper.

## Discussion

There is limited understanding of the mechanism behind steroid-induced mania, but it is thought to resemble the pathophysiology behind Cushing’s disease and other disorders of the hypothalamic-pituitary-adrenal (HPA) axis [7]. In the body, cortisol activates both mineralocorticoid and glucocorticoid receptors. Exogenous steroids, including synthetic corticosteroids such as prednisone, preferentially activate glucocorticoid over mineralocorticoid receptors [8]. In addition, exogenous steroids provide negative feedback to the HPA axis and suppress endogenous cortisol secretion from the adrenal glands. Consequently, there is less cortisol available to activate mineralocorticoid receptors, and thus, there is increased glucocorticoid receptor stimulation compared to mineralocorticoid receptor stimulation. This imbalance results in cognitive impairment and emotional disturbances as observed in individuals with corticosteroid-induced mania [8,9].

Psychiatric reactions to steroid treatment are typically seen in patients receiving high-dose systemic steroids. Fewer than 2% of patients that developed neuropsychiatric symptoms (hallucinations, delusions, violent behavior, inappropriate euphoria, depression, mania) on steroids were receiving doses less than 40 mg per day of prednisone or its equivalent [6,10]. In contrast, approximately one in five individuals on 80 mg or more per day of prednisone develop such symptoms [6]. Therefore, it is unusual that this patient developed manic symptoms while on a dose of 30 mg per day. While the patient was on higher doses in the past that may have triggered these symptoms, it is much more common for patients to develop psychiatric symptoms immediately after starting the medication, with 60-85% of patients developing symptoms within the first week of receiving steroids [6]. At the onset of her symptoms, the patient had taken less than 40 mg prednisone per day for almost a week. Additionally, she had previously been on doses of 40 mg or higher for approximately three weeks without neuropsychiatric symptoms. However, there is no proven correlation linking previous tolerance of corticosteroids with a decreased incidence of developing neuropsychiatric symptoms when restarting the medication [6]. Factors that may have increased her risk for developing such neuropsychiatric symptoms include having SLE and being female [6].

Given the response observed in this patient, caution should be taken when reintroducing patients to steroids, even if they will be receiving doses lower than they previously tolerated. Prescribers should also be aware of the signs of corticosteroid-induced mania. Beyond the classic symptoms of mania such as pressured speech, increased activity, and agitation, patients with mania specifically due to steroids are more likely to exhibit psychotic symptoms including persecutory delusions, auditory hallucinations, and disorganized behavior [6]. Therefore, evaluation should include symptoms of both mania and psychosis.

Limitations of this case report include restricted follow-up of the patient to assess for further psychiatric symptoms and the possibility of an alternate diagnosis. Other diagnoses considered in this patient were meningitis and neuropsychiatric manifestations of lupus. While workup was largely unremarkable and most suggestive of corticosteroid-induced mania, the cerebrospinal fluid analysis did show a lymphocytic pleocytosis and aseptic meningitis was not definitively ruled out. This patient’s quick resolution of symptoms following initiation of valproic acid makes aseptic meningitis a less likely etiology. Given the overlapping symptoms of neuropsychiatric manifestations of lupus and corticosteroid-induced mania, it is possible this patient’s symptoms could be attributed to lupus [3]. Typically, patients will exhibit other symptoms of lupus exacerbations in addition to neuropsychiatric ones, including cutaneous, articular, and hematological involvement [3]. In addition, the neuropsychiatric manifestation of lupus is more characteristically psychosis, cognitive dysfunction, peripheral neuropathy, and cerebrovascular disease rather than symptoms of mood disorder, especially mania [3]. As this patient was not reporting or exhibiting signs of arthralgias, rashes, and oral sores and her neuropsychiatric symptoms were limited to mania, her presentation is more consistent with corticosteroid-induced mania.

## Conclusions

This case report described a patient who previously tolerated high doses of corticosteroids without experiencing neuropsychiatric symptoms but later developed corticosteroid-induced mania upon restarting therapy. Although research suggests a dose-dependent relationship between steroids and neuropsychiatric side effects, previous tolerance of steroids has not been found to be associated with decreased frequency of corticosteroid-induced mania upon restarting the medication. Thus, patients should be monitored for such side effects not only on initiation but also on the reintroduction of steroid therapy.
